# Parent and Child’s Negative Emotions During COVID-19: The Moderating Role of Parental Attachment Style

**DOI:** 10.3389/fpsyg.2021.567483

**Published:** 2021-03-05

**Authors:** Ziqin Liang, Elisa Delvecchio, Yucong Cheng, Claudia Mazzeschi

**Affiliations:** Department of Philosophy, Social Sciences and Education, University of Perugia, Perugia, Italy

**Keywords:** attachment style, negative emotion, emotion regulation, COVID-19, children, parent

## Abstract

In February 2020, the coronavirus disease 2019 (COVID-19) appeared and spread rapidly in Italy. With the health emergency and social isolation, parents started spending more time with their children, and they might have experienced greater distress. Attachment style is considered as an effective emotion regulation strategy in the parent–child relationship. However, few empirical studies have addressed this issue. Based on attachment theory, this study aimed to find parental attachment style as a candidate to moderate the relation between parents’ negative emotions and their perceptions of their children’s negative emotions related to COVID-19. Parents (*M*age = 42.55 ± 6.56, 88.2% female) of 838 Italian children and adolescents aged 3 to 18 years participated in an online survey. Results showed that parents with a fearful attachment style had significantly higher negative emotions when facing COVID-19 than those with other attachment styles. Moreover, parents with a dismissing attachment style perceived fewer negative emotions in their children than parents with fearful and preoccupied styles. At last, higher parents’ negative emotions were associated with greater perception of children’s negative emotions only in parents classified as secure and fearful. These findings suggest that parents with dismissing and fearful attachment styles and their children may be at higher risk during the COVID-19 pandemic and they should be given long-term attention.

## Introduction

In February 2020, the coronavirus disease 2019 (COVID-19) spread rapidly in Italy and then forced a comprehensive lockdown on March 10, 2020. The whole country entered a large-scale isolation stage for the first time to control the pandemic (Liang et al., 2020). Together the health emergency and social isolation has caused great psychological pressure and several daily life changes for individuals.

In a recent survey of Chinese adults, it was found that individuals’ perceived severity of COVID-19 increased their negative emotions (Li Y. et al., 2020), with higher levels of stress, anxiety, depression, and other symptoms (Wang H. et al., 2020). A survey with adults aged 16–75 years in the United Kingdom also showed that social isolation and loneliness brought on by COVID-19 increased the risk of anxiety, depression, and other negative consequences (Holmes et al., 2020). Similar results were also found in other countries (Moccia et al., 2020; Odriozola-González et al., 2020; Orgilés et al., 2020). In addition to adults, studies have shown that even young children can be aware of changes in their surroundings (Dalton et al., 2019, 2020). In a survey of Italian and Spanish parents, they realized that some of their children’s emotions (such as boredom and irritability) and behaviors (such as sleep time) had changed compared with those before isolation (Orgilés et al., 2020).

During the isolation period, most people had to stay with their families, parents and children spent more time with each other, and they might have experienced greater distress. Adults’ attention, reactions, and emotions toward COVID-19 affect their ability to sensitively recognize their children’s troubles (Dalton et al., 2020). Research suggests that regardless of the child’s age and gender, children and adolescents adjust their own emotions and behaviors according to adults’ emotions and reactions (Stein et al., 2009; Xu et al., 2020). They are more likely to experience the pain, fear, and anxiety of those around them than adults (Bartlett et al., 2020). They would display more negative emotions and problem behaviors than usual (Liang et al., 2020). Previous studies on the impact of major disasters or sudden public health events on children’s mental health have emphasized that children’s negative emotions in different degrees may be due to the negative emotions of their parents (Sprang and Silman, 2013; Juth et al., 2015; Cobham et al., 2016). Unexplained and unpredictable situations such as the COVID-19 pandemic and the time of quarantine might be perceived as threatening and stressful by adults leading to negative emotions such as anxiety. Thus, when parents showed more negative emotions than those before isolation, children and adolescents were likely to be affected and might be at higher risk for psychological maladjustment (Dalton et al., 2020).

Parents are an important part of a child’s personal resources, which can provide resilience-related factors to alleviate children’s mental health problems in particularly stressful situations (Holmes et al., 2020). Parents, as a result of spending more time with their children and adolescents during lockdown, are their main and most available support. Previous findings suggest that parental stress is a powerful predictor of children’s post-disaster adjustment (Pfefferbaum et al., 2015). Parents under a lot of pressure can be detrimental to a child’s recovery from a disaster. The outbreak of the COVID-19 pandemic and the subsequent implementation of social isolation measures are not only inconvenient to life and daily routines, but also cause most parents to undertake a conversion in their family (parent) and work (employee) roles, in which, the boundary between family and work becomes challenging (Restubog et al., 2020). In the face of such changes, parents bear high levels of stress, anxiety, and other negative emotions (Sang et al., 2019; Li S. et al., 2020; Pfefferbaum and North, 2020). Therefore, paying attention to parents’ reactions and emotions can help find children and adolescents who are at a higher risk for psychological maladjustment during COVID-19, and plan specific interventions in the long term.

Parents’ attachment style is one of the crucial factors connecting parents and their children (Li et al., 2015; Schimmenti and Bifulco, 2015). In times of stress, such as in the face of the pressure of COVID-19, attachment style may be seen as one of the key factors contributing to the emotion regulation of parents and children (Shaver and Mikulincer, 2007; Mikulincer and Shaver, 2019). Individuals with different attachment styles have different emotional reactions and ways of dealing with negative and positive events in life. Bartholomew and Horowitz (1991) proposed a four-category attachment model: secure, dismissing, fearful, and preoccupied. Secure attachment can be used as a flexible resource when needed, and it is the foundation of an individual’s mental health and social adaptation (Mikulincer and Shaver, 2019). People with secure attachment style are more likely to effectively regulate their negative emotions and have hope for solving problems when they experience fear and threats (Nielsen et al., 2017). On the other hand, insecure adults have difficulties in emotion regulation (Jones et al., 2014). Dismissing, preoccupied, and fearful attachment styles are all seen as insecure, and they exhibit a higher level of anxiety and avoidance than secure attachment (Bartholomew and Horowitz, 1991; Welch and Houser, 2010). In detail, those with preoccupied and fearful attachment styles show stronger negative emotional reactions when facing negative events (Gentzler et al., 2010). Insecure attachment strategies will repeatedly activate and suppress negative emotions, and continue to rely on the distorted self and others, leading to poor physical and mental health (Mikulincer and Shaver, 2019; Pace et al., 2019).

Parents differ in their ability to communicate, feel, and respond to their children’s emotions based on their attachment style (Sloman et al., 2002). Parents with a secure attachment style can provide emotional feedback to their children and respond to children’s various emotions in a consistent and sensitive manner, so that children can adjust their emotional experience and promote the development of emotion regulation ability, whereas parents with insecure attachment styles provide less supportive and constructive behavior (Feeney and Collins, 2001; Jones et al., 2015). For example, parents with dismissing attachment style show limited ability to express emotions, and they show withdrawal if there is negative influence in the interaction with their children. Children of such parents adopt unsafe and avoidant emotion regulation strategies to minimize the expression of their own emotions, in order to get as close to the caregiver as possible (Sloman et al., 2002). Fearful parents have low trust in others and have a certain reaction to their children’s emotional needs, but they keep a distance from them and avoid intimate contact with children to protect themselves from the expected rejection of others (Kilmann et al., 2009). Parents with a preoccupied attachment style have no goals to express themselves; they respond differently to their children and provide few emotional anchors to assist in regulating their emotional state. Children of such parents cannot predict their parents’ reactions and will develop greater insecurity and anxiety (Sloman et al., 2002). Mothers who reported greater attachment-related avoidance and anxiety had greater difficulties in regulating their emotions, resulting in more painful and less supportive responses to their children’s negative emotions (Jones et al., 2014). Surprisingly, children with dismissing parents would report that their parents are warm and caring (Borelli et al., 2013). Researchers suggest that individuals with dismissing attachment style will not only evaluate stressful events as threatening but also evaluate themselves as able to cope with stressors (Mikulincer and Shaver, 2007). They have higher resilience and have a positive effect on well-being (Karreman and Vingerhoets, 2012). It may be because they have a higher degree of avoidant behaviors (Bartholomew and Horowitz, 1991; Wei et al., 2011); when encountering stressful events, they preferably use distancing coping strategies (such as stress denial) to maintain distance to others (Karreman and Vingerhoets, 2012). They may develop a survival tool of compulsively relying on themselves because of their caregivers’ rejection and unresponsiveness (Wei et al., 2011). They often avoid or do not express their distress and idealize their parent–child relationship or tend to evaluate it positively (Borelli et al., 2013). However, such individuals may lack the correct understanding of themselves (Wei et al., 2011). Children whose parents were unresponsive to their needs would tend to, as adults, deactivate their attachment system in order to repress their emotions and withdraw from intimate relationships (Mikulincer et al., 2003). Thus, children with dismissing parents seem to be worthy of attention, because they would have resilience when dealing with short-term stress events, but if they were under long-term stress, they may be in trouble in later development (Bowlby, 1969).

Recent research suggests that COVID-19 and its consequences represented a risk factors for several kids (Holmes et al., 2020; Jiao et al., 2020; Lee, 2020; Liang et al., 2020; Orgilés et al., 2020; Wang G. et al., 2020). The long duration of isolation, fear of infection, frustration and boredom, lack of face-to-face contact with classmates, friends, and teachers, and the lack of personal space at home and other stress factors have caused children and adolescents to display varying degrees of negative emotions (Brooks et al., 2020; Wang G. et al., 2020). According to a survey of children and adolescents aged 3–18 years in China, which was completed by the parents, stress, distraction, and restlessness were the most common psychological and behavioral problems during isolation (Jiao et al., 2020). Similar results have been found in survey of parents with children aged 3 to 18 years old from Italy and Spain (Orgilés et al., 2020).

A recent survey on the psychological impact of COVID-19 showed that adults’ attachment style modulated stress responsivity, and contrary to secure individuals, insecure individuals had a weaker ability to regulate emotion (Moccia et al., 2020). Furthermore, empirical studies showed that, in the face of disaster, supportive parenting behaviors can reduce children’s anxiety (Pfefferbaum et al., 2015). Parents with secure attachment style have fewer conflicts with their children (La Valley and Guerrero, 2010), and it is negatively related to adolescents’ emotional problems, such as depressive symptoms (Zhang et al., 2010; Li et al., 2015). Therefore, it can be inferred that, under the influence of the COVID-19 pandemic, parents would experience different degrees of negative emotions due to their different attachment, among which, parents with secure attachment would have the lowest negative emotions compared to others, while parents with fearful attachment would display the most serious negative emotions. Moreover, parents reporting higher negative emotions were expected to depict children and adolescents with more negative emotions. Furthermore, it was hypothesized that parents’ attachment style moderated the relation between parents’ negative emotions and children’s negative emotions as perceived by their parents. In detail, parents with a secure attachment style would report a lower level of negative emotions in their children than parents with insecure attachment. Dismissing parents would perceive a lower level of negative emotions in their children due to the habit of covering up their negative emotions, while parents with fearful and preoccupied attachment styles would describe children as having a higher level of negative emotions due to their less effective parenting behaviors linked to their own negative emotional reaction.

## Materials and Methods

### Participants and Procedures

Between March 26 and April 5, 2020, parents of 838 Italian children and adolescents participated in our online survey. The children’s ages ranged from 3 to 18 years old (*M*age = 9.65, *SD* = 4.36, 49.4% female). Table 1 lists the sample characteristics. Parents had a mean age of 42.65 years (*SD* = 6.49, 88.3% female). In total, 69.9% had more than one child. Almost all participants (97.1%) had an Italian nationality, most of them had a monthly family income of more than 2,000 Euros (60.8%), and 57.8% of them held a bachelor degree or above, suggesting that a majority of the families came from a middle class context (i.e., SES level 3; Hollingshead, 1975).

**TABLE 1:** Sample characteristics in sociodemographic variables (*N* = 838).

Variables	*N*	*%*
**Parents**		
Mother	740	88.3
Age, *M* (*SD*)	42.65	6.49
**Monthly family income (Euros)**		
Up to 999	43	5.1
Between 1000 and 1999	186	22.2
Between 2000 and 3999	240	28.6
Between 3000 and 4999	206	24.6
5000 or more	64	7.6
**Education level**		
Primary school	39	4.7
Secondary school	315	37.6
Undergraduate	357	42.6
Doctoral or master	127	15.2
**Mother’s current employment situation**		
Self-employed	129	15.4
Part-time	153	18.3
Full-time	216	25.8
Unemployed	53	6.3
Lost job due to COVID-19	41	4.9
Smart-working	178	21.2
Other	55	6.6
**Father’s current employment situation**		
Self-employed	225	26.8
Part-time	19	2.3
Full-time	399	47.6
Unemployed	16	1.9
Lost job due to COVID-19	17	2.0
Smart-working	134	16.0
Other	4	0.5
**Children**		
Female	411	49.4
Age, *M* (*SD*)	9.65	4.36

This study was approved by the Ethical Committee for psychological research by the authors’ university. Due to quarantine constraints, school principals and/or social networks (e.g., WhatsApp groups) were used to send out emails, using a snowball sampling strategy, to invite parents to join in the study. Informed consent was obtained before participants filled in all the questions voluntarily and anonymously, and it took about 10 min to complete the survey. Only one parent per household was required to participate in the study. Parents with more than one child were asked to fill in the questionnaire for each child.

### Measures

#### Parental Attachment Style

The Italian version of the Relationship Questionnaire (RQ) was used to measure adult attachment style (Fossati et al., 2007; Ravitz et al., 2010). This questionnaire includes two sections in which participants are required to evaluate: (a) four short essays describing the prototypes of the four attachment styles in the attachment process, which are secure, fearful, preoccupied, and dismissing; (b) their degree of conformity to each essay with a 7-point scale. For the present work, it was administered only for the first section. RQ showed adequate psychometrics characteristics (Ruvolo et al., 2001; Cozzarelli et al., 2003).

#### Parents’ Negative Emotions

Parents’ negative emotions during the COVID-19 pandemic were measured with the Impact Scale of COVID-19 and home confinement (Orgilés et al., 2020). This scale includes three items rated on a 5-point Likert scale (from 1 = *nothing serious* to 5 = *very serious*). A higher score indicates that participants’ negative emotions when facing COVID-19 are more severe. Sample items include: “How afraid are you of losing your job?” The Cronbach’s α was 0.539 in this study.

#### Children’s Negative Emotions

Children’s negative emotions were measured by the Impact Scale of COVID-19 and home confinement on children and adolescents (Orgilés et al., 2020). This scale includes 31 items of children’s psychological responses to quarantine with a 5-point scale (from 1 = *Much less* to 5 = *Much more*). For the present work, parents reported on 18 items related to children’s negative emotions based on their observations. A higher score indicates that participants’ perception of children’s negative emotions when facing the COVID-19 are more severe. Sample items include “Is afraid about COVID-19 infection” and “Is worried”. The Cronbach’s α was 0.931 in this study.

### Data Analysis

Statistical Package for Social Science (IBM SPSS Version 21) and R Studio (Version 3.6.2) were used for data analysis. First, we carried out descriptive statistics to describe the data of the present sample. Second, correlation analysis and a series of analyses of variance (ANOVAs) were carried out to examine the association among the variables. Effect size was measured using partial eta-squared, in which small, medium, and large effects were 0.0099, 0.0588, and 0.1379, respectively (Cohen, 1988, p. 283). The Bonferroni *post hoc* test was used to compare means. Finally, hierarchical regression models were used to examine the moderation effect of attachment style in the association between parents’ negative emotions and children’s negative emotions. In the case of significant interaction effects, simple slope analysis was completed to examine the effects of parents’ negative emotions separately by attachment styles. Given that attachment styles were multi-categorical variables representing four groups, three dummy variables (R1, R2, and R3) were created, with dismissing attachment serving as the reference group (dismissing: R1 = 0, R2 = 0, R3 = 0; fearful: R1 = 1, R2 = 0, R3 = 0; preoccupied: R1 = 0, R2 = 1, R3 = 0; secure: R1 = 0, R2 = 0, R3 = 1).

## Results

### Descriptive Statistics, Correlations, and ANOVAs

Parents perceived more negative emotions (*M* = 3.04 ± 0.781) when they faced COVID-19, and they noticed an increase in children’s negative emotions compared to before quarantine (*M* = 2.90 ± 0.726).

Correlation analysis showed that parents’ negative emotions were significantly positively related to perceived children’s negative emotions (*r* = 0.135, *p* < 0.01).

The results of the ANOVAs showed that a main effect was found on parents’ negative emotions depending on different attachment styles (*F*_(__3_,_834__)_ = 6.71, *p <* 0.001, *ŋ_*p*_^2^* = 0.024). *Post hoc* analyses revealed that parents with a fearful attachment style had significantly higher negative emotions when facing COVID-19 than those with other attachment styles. Children’s negative emotions perceived by their parents also showed a significant effect depending on different attachment styles (*F*_(__3_,_834__)_ = 4.22, *p <* 0.01, *ŋ_*p*_^2^* = 0.015). Children with dismissing parents were perceived to display significantly lower negative emotions than those with parents with a fearful and preoccupied attachment style (see Table 2).

**TABLE 2:** Parents’ negative emotions and children’s negative emotions according to attachment styles and *post hoc* comparisons.

	Secure (1)		Fearful (2)		Preoccupied (3)		Dismissing (4)		*F*	Effect	*Post hoc*
	(*n* = 385)		(*n* = 172)		(*n* = 109)		(*n* = 172)			size (*ŋ_*p*_^2^)*	
	*M*	*SD*	*M*	*SD*	*M*	*SD*	*M*	*SD*			
Parents’ negative emotions	2.96	0.77	3.27	0.72	3.00	0.78	2.99	0.81	6.71***	0.024	2 > 1,3,4
Children’s negative emotions	2.90	0.73	2.97	0.67	3.02	0.68	2.75	0.76	4.22**	0.015	4 < 2,3

**p < 0.01; ***p < 0.001.

### The Moderation of Attachment Styles in the Association Between Parents’ Negative Emotions and Children’s Negative Emotions

We conducted hierarchical regression models to examine the association between parents’ negative emotions and children’s negative emotions as well as the moderation of attachment styles. The results of the full model are summarized in Table 3. The results showed that the main effect of parents’ negative emotions on children’s negative emotions was significant, suggesting that parents who perceived COVID-19 to be more severe observed more negative emotions in their children (see Model 1). The main effect of different attachment styles on children’s negative emotions were also significant (see Table 3, Model 2). Parents with fearful, preoccupied, and secure attachment styles depicted their children with higher negative emotions than parents classified as dismissing. Furthermore, looking at Model 3, results showed that the interaction effects between parents’ negative emotions and attachment styles were also significant.

**TABLE 3:** Hierarchical regression model of the association between parents’ negative emotions and children’s negative emotions and the moderation effect of attachment styles.

		*B*	*SE*	*R*^2^	Adjusted *R*^2^	*F*
Model 1	Parents’ negative emotions	0.13***	0.03	0.018	0.017	15.54***
Model 2	Parents’ negative emotions	0.12***	0.03	0.032	0.027	6.789***
	R1	0.19*	0.08			
	R2	0.27**	0.09			
	R3	0.16*	0.07			
Model 3	Parents’ negative emotions	−0.05	0.07	0.042	0.034	5.22***
	R1	0.18*	0.08			
	R2	0.28**	0.09			
	R3	0.17*	0.07			
	Parents’ negative emotions × R1	0.26**	0.10			
	Parents’ negative emotions × R2	0.21	0.11			
	Parents’ negative emotions × R3	0.22***	0.08			

R1, R2, R3 were dummy variables for coding attachment styles. Dismissing: R1 = 0, R2 = 0, R3 = 0; fearful: R1 = 1, R2 = 0, R3 = 0; preoccupied: R1 = 0, R2 = 1, R3 = 0; secure: R1 = 0, R2 = 0, R3 = 1. *p < 0.05;**p < 0.01; ***p < 0.001.

**FIGURE 1:**
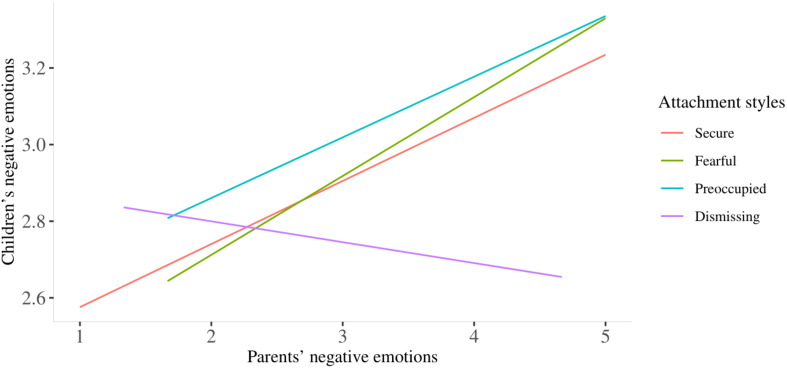
The association between parents’ negative emotions and children’s negative emotions by attachment styles.

Simple slope analysis revealed that the association between parents’ negative emotions and perceived children’s negative emotions had a significant positive slope in both the fearful and secure group (see Figure 1). In other words, higher parents’ negative emotions were associated with greater perception of children’s negative emotions only in parents with secure and fearful attachment styles. No significant effects were found referring to dismissing and preoccupied ones.

## Discussion

COVID-19 has spread rapidly around the world, and the scope and extent of its impact was unexpected (Brooks et al., 2020; Holmes et al., 2020; Liang et al., 2020; Pfefferbaum and North, 2020). The current study aimed to explore how negative emotions observed in parent–child dyads in normative situations could assume a central role during not-normative situations such as the COVID-19 pandemic. In particular, parental attachment style appeared as a possible risk or resilience factor for emotional regulation of negative feelings showed by parents and perceived in their children during the pandemic.

This study found that compared with the emotions before isolation, both parents and children showed higher negative emotions during the isolation period. Parents’ negative emotions affected children’s negative emotions, when parents showed more negative emotions, their children were perceived as showing more negative emotions. It was consistent with our hypothesis, and previous studies which have shown the same results, indicating that the performance of parents’ emotion regulation affects their children’s emotion regulation in particularly stressful situations (Sprang and Silman, 2013; Juth et al., 2015; Pfefferbaum et al., 2015; Cobham et al., 2016; Holmes et al., 2020; Liang et al., 2020; Orgilés et al., 2020).

In addition, more importantly, it was found that the attachment style moderated the association between parents’ negative emotions and children’s negative emotions. When parents showed greater negative emotions, they could observe that children with parents with a dismissing attachment style reduced their negative emotions. According to the four-category attachment model (Bartholomew and Horowitz, 1991), parents with a dismissing attachment style find it difficult to trust or rely on others, so they often adopt negative parenting strategies when facing their children. Previous studies have also shown that dismissing individuals have less close and less satisfying relationships (Bartholomew and Horowitz, 1991; Wei et al., 2011; Tan et al., 2012). If parents and children are not used to expressing their emotions to each other, then children are more likely to control or cover up, deliberately underestimate, or even defensively deny the presence of their negative feelings without showing their true emotions in front of their parents (Sloman et al., 2002). Such children might be at higher risk because their parents cannot recognize their true feelings and have a weak sense of their emotional state (Brenning et al., 2012).

In contrast, parents with a fearful attachment style showed more negative emotions, and in turn, their children were reported as displaying more negative emotions as well. Parents classified as fearful harbor a negative view of the self, showing a strong sense of dependence on others and high anxiety. In the interaction activities between parents and children, parents may question their own value and worry about losing the child, so they will pay too much attention to the child and constantly monitor the child’s emotions, but sometimes they are more focused on their own relational needs and worries of being rejected than on their children’s emotional state. It may exacerbate the child’s negative experience (Sloman et al., 2002). Under the pressure of COVID-19, these children may not only bear the worry and pressure of the pandemic but also bear the parents’ maladjusted emotions and reactions, thus exacerbating the children’s negative emotional burden. Unexpectedly, the perception of children’s negative emotions by secure parents was also more severe with the increase of their own negative emotion. This may be because secure parents have close relationships with their children, and their children often calm themselves and increase feelings of safety by seeking proximity to parents (Esbjørn et al., 2012). This might allow parents to have a more comprehensive and accurate perception of their child’s emotions. In the face of the pandemic situation, when parents’ emotional performance was more negative, children would feel the same strong severity, so they would be more negative. However, compared with the fearful attachment style, secure parents and their children displayed lower levels of negative emotions, it might be because in the daily activities between parents and children, the supportive parenting behaviors promote the development of the children’s emotional regulation ability (Jabeen et al., 2013; Jones et al., 2015; Shlafer et al., 2015). Under the influence of the pandemic, they could regulate their emotions better.

The results of this study indicate that, in the face of major disasters or sudden public health events, attachment style plays a key role in moderating the relationship between parents and children. These findings offer important implications in understanding and improving the mental health of children and adolescents under the influence of COVID-19. Parents with secure attachment can regulate their emotion under stressful situations, and their children will also be affected by them. Parents should provide more supportive parenting behaviors to promote children’s emotional regulation ability. Parents with insecure attachment have a higher degree of adverse adaptation reactions under stressful and threatening events, and at the same time, their children may also experience negative emotions. These children may need more attention in the long term. In particular, the dismissing attachment style can buffer the association between parents’ and children’s emotions, but these kind of children may suppress their own emotions or their emotions have been ignored by their parents. Therefore, parents and children dyads characterized by dismissing and fearful parents might need long-term attention in order to monitor and support their negative emotions and their intrapersonal and interpersonal psychological adjustment.

It should be pointed out that there are still some limitations in this study. First, due to the limitation of pandemic isolation and considering the children’s cognitive levels and comprehension, the information was only reported by parents, which increases common method bias. Moreover, the cross-sectional nature prevents us from inferring causality. Future research should collect self-reports from children and further reveal a causal relation through longitudinal design. In addition, some populations were over-represented, such as mothers. Future studies should collect a wider variety of representative samples in order to achieve more robust findings. Third, only three items were used to measure the parents’ negative emotions, which may have made the internal consistency reliability of the scale not as high as desired. In addition, future research can explore other variables possibly affecting parents’ emotions, and the perceptions of their children’s feelings such as emotion regulation (Pace et al., 2018), alexithymia (Pace et al., 2015), and reflective functioning (Schultheis et al., 2019). Furthermore, future studies should also take into account children’s attachment and they should explore in-depth the direction of the relation between parental negative emotions and children’s negative emotions. Despite these limitations, this study could be the first applying the knowledge of the normative processes of parent–child emotion regulation, mediated by parental attachment style, in a not-normative situation like COVID-19. Moreover, the findings can be useful to support how certain processes, highlighted in non-risk situations, are applicable and take on particular relevance in planning family support and interventions in critical situations such as the current pandemic.

## Data Availability Statement

The raw data supporting the conclusions of this article will be made available by the authors, without undue reservation.

## Ethics Statement

The studies involving human participants were reviewed and approved by the Ethics Committee of the Department of Philosophy, Social Sciences and Education of the University of Perugia (Italy). The patients/participants provided their written informed consent to participate in this study.

## Author Contributions

ZL, ED, YC, and CM contributed to the conception and design of the study. ZL and YC organized the database and performed the statistical analysis. ZL and ED wrote the first draft of the manuscript. CM wrote the sections of the manuscript. All authors contributed to manuscript revision, and read and approved the submitted version.

## Conflict of Interest

The authors declare that the research was conducted in the absence of any commercial or financial relationships that could be construed as a potential conflict of interest.
